# Protecting Future Children from In‐Utero Harm

**DOI:** 10.1111/bioe.12238

**Published:** 2016-02-12

**Authors:** Dominic Wilkinson, Loane Skene, Lachlan De crespigny, Julian Savulescu

**Keywords:** medical ethics, pregnancy, fetus, fetal alcohol spectrum disorders, law

## Abstract

The actions of pregnant women can cause harm to their future children. However, even if the possible harm is serious and likely to occur, the law will generally not intervene. A pregnant woman is an autonomous person who is entitled to make her own decisions. A fetus in‐utero has no legal right to protection. In striking contrast, the child, if born alive, may sue for injury in‐utero; and the child is entitled to be protected by being removed from her parents if necessary for her protection. Indeed, there is a legal obligation for health professionals to report suspected harm, and for authorities to protect the child's wellbeing. We ask whether such contradictory responses are justified. Should the law intervene where a pregnant woman's actions risk serious and preventable fetal injury? The argument for legal intervention to protect a fetus is sometimes linked to the concept of ‘fetal personhood’ and the moral status of the fetus. In this article we will suggest that even if the fetus is not regarded as a separate person, and does not have the legal or moral status of a child, indeed, even if the fetus is regarded as having no legal or moral status, there is an ethical and legal case for intervening to prevent serious harm to a future child. We examine the arguments for and against intervention on behalf of the future child, drawing on the example of excessive maternal alcohol intake.

## Introduction

Pregnancy can lead to unique legal and ethical challenges. A pregnant woman is an autonomous person who is entitled to make her own decisions. In most jurisdictions, the fetus in‐utero has no legal right to protection. While the actions of pregnant women can cause harm to their future children, even if the possible harm is serious and likely to occur, the law will generally not intervene. In striking contrast, the later child, if born alive, may sue for injury in‐utero under the Congenital Disabilities Act 1976; and the child is entitled to be protected by being removed from her parents if necessary for her protection. Indeed, there is a legal obligation for health professionals to report suspected harm, and for authorities to protect the child's wellbeing. Are such contradictory responses justified? Should the law intervene where a pregnant woman's actions risk serious and preventable fetal injury?

The argument for legal intervention to protect a fetus is sometimes linked to the concept of ‘fetal personhood’ and the moral status of the fetus. In this article we will suggest that even if the fetus is not regarded as a separate person, and does not have the legal or moral status of a child, indeed, even if the fetus is regarded as having no legal or moral status, there is an arguable ethical and legal case for intervening to prevent serious harm to a future child.^1^ We examine the arguments for and against intervention on behalf of the future child, drawing on the example of excessive maternal alcohol intake.

## In‐Utero Harm

Birth defects occur in approximately 3% of live‐born babies.^2^ Some of these have genetic causes. However, in many cases of congenital abnormality, there is no identifiable genetic abnormality, and some defects are likely to be related to environmental factors impacting on the developing fetus.^3^ A very large number of agents, activities and factors have been associated with in‐utero harm.

There are several ethically significant features of in‐utero harm. First, the rapid development taking place in fetal life means that relatively small interventions or influences can have profound, life‐long effects. Second, the range of different factors that can cause harm can make it very difficult (in retrospect) to identify a single factor as causative of harm in individual cases. Third, harm to a fetus in‐utero occurs at a time of contested (and, on some accounts, reduced) moral status.

In‐utero harm could be classified into different categories. Some in‐utero harm occurs as a result of the action of third parties. For example, third party harm resulting from physical assault to a pregnant woman, or the teratogenic effects of a drug that has been negligently prescribed or inadequately evaluated. In‐utero harm can also occur as a result of the action of the pregnant woman herself (call this “*gestational harm”*). In this article we will focus mostly on gestational harm, however, it will be useful to briefly consider third party harm. In‐utero harm might also be divided into lethal (leading to miscarriage or in‐utero fetal death) and non‐lethal forms. We will focus largely on non‐lethal in‐utero harm.

There are many situations in which a pregnant woman's actions could seriously but non‐lethally harm her fetus and future child. One of the most widely cited is the link between drinking alcohol during pregnancy and fetal alcohol spectrum disorders (FASD) (Box 1). There may also be preventable damage when pregnant women use other substances including illicit drugs and tobacco. In the United States, an estimated 4.4% of pregnant women apparently reported illicit drug use in the 30 days before a study undertaken in 2010.^4^

Box 1 **Non‐lethal gestational harm. The example of Fetal Alcohol Spectrum Disorders**

Fetal Alcohol Spectrum Disorders (FASD)

Alcohol consumption during pregnancy can result in a range of fetal abnormalities including abnormal facial features, problems in growth and damage to the developing central nervous system.^5^ Heavy exposure to alcohol is consistently associated with intellectual deficit, with most children and adults with FASD having mild‐borderline intellectual disability. One prospective study identified a dose‐dependent effect, with each ounce of alcohol/day associated with a 5 point decrease in full‐scale IQ.^6^ Affected children often have associated reductions in attention and executive functioning,

FASD is the most common preventable cause of brain damage in newborn infants.^7^ Alcohol‐related harm is estimated to affect 1% of births.^8^ These disabilities cause life‐long injury, which may severely undermine the welfare of the affected child and adult. Globally, more than 1 million babies each year are born with preventable, permanent brain injury. In a recent study, one in eight children born in a remote Australian community were diagnosed with FASD.^9^

It is difficult to determine the absolute risk of FASD for a particular amount of alcohol consumption.^10^ It is often stated that there is no safe level, though systematic reviews have not clearly identified deficits with moderate levels of consumption.^11^ Various factors (apart from the amount of alcohol consumed) appear to influence the risk. In the recent Australian study, 25% of children whose mothers had drunk alcohol at high‐risk levels during pregnancy were diagnosed with FASD.

## Third Party In‐Utero Harm and Fetal Personhood

Where in‐utero harm has been caused by a third party, there is a strong intuitive case that the third party should be subject to legal sanction, and that the parents and child should be legally entitled to financial compensation. Consider the following cases:

*The non‐lethal teratogen*. Company X releases a new drug for morning sickness. Its own research suggests that this drug is associated with a 25% chance of causing physical defects and brain damage to fetuses. Nevertheless, the company conceals this research and releases the drug to the market. Twenty‐five percent of pregnant women taking the drug subsequently have a fetus affected by physical defects and brain damage

*The lethal teratogen*. Company Y releases a new drug for morning sickness. Its own research suggests that this drug is associated with a 25% chance of causing miscarriage. Nevertheless, the company conceals this research and releases the drug to the market. Twenty‐five percent of pregnant women taking the drug subsequently miscarry

Should the children and parents affected by the teratogen cases be entitled to legal redress?

Traditionally, compensation has been available to parents for pre‐birth injury to their child after birth in ‘non lethal’ cases but not where the injury is caused to a fetus which dies in‐utero.^12^ In the latter situation, compensation can only be claimed ‘indirectly’ by the woman for the loss of the pregnancy, not for any injury to her child. A claim relating to a child's pre‐birth injury can be made by the parents only if the child is born alive.^13^

This issue is fraught with controversy, as was evident in discussion arising from a Bill for ‘Zoe's law’^14^ that was proposed in New South Wales, Australia after a court case involving an accident that caused a pregnant woman, Ms Brodie Donegan, to suffer a 32‐week fetal death. The driver at fault was prosecuted and convicted of having caused grievous bodily harm to Ms Donegan. However, the law did not provide for a separate offence or penalty in respect of the fetus, Zoe. Ms Donegan believed that this omission meant that Zoe's death was not recognized by the law.^15^ The aim of the Bill (which lapsed in the upper house) was to enable a person who has caused the death of a fetus of a least 20 weeks gestation to be charged with causing grievous bodily harm to the fetus as well as to the pregnant woman through the loss of the pregnancy.^16^ That proposal was widely criticized. In particular, it was argued that regarding a fetus as a person separate from the woman could lead to restrictions on the reproductive choices and behaviour of pregnant women;^17^ and this, in turn, would have implications for the law of abortion. Those concerns were evident in commentary and debates when the Bill for ‘Zoe's law’ was proposed.^18^ A woman's right to terminate a pregnancy, including an early pregnancy, might be more readily challenged if she is regarded as killing a separate ‘person’ rather than doing an act that affects her own body; or killing a person rather than preventing one coming into existence. This is not a new issue, of course, but it is one that raises difficult and divisive issues in the community. It may be better not to conceptualize a fetus as a ‘person’, even if one takes a ‘mid‐way’ view of the relationship between woman and fetus, for example, ‘not one, but not two’.^19^

## Third Party Harm and the Future Child

On the other hand, if the child is not regarded as a ‘person’ when the harm was committed there is a difficult question about whether there is any legal redress possible in either of the teratogen cases. How can harm be caused to someone who is not a person? Can a non‐person be the victim of a crime? Under Australian law, after the child's live birth, the parents can claim compensation relating to a pre‐birth injury (such as their costs in raising the child to adulthood), but the child cannot claim on their own behalf for compensation for their pre‐birth injuries.^20^

One option is to draw upon a different conceptualization of the fetus. Instead of focusing on the injury at the time the harm was caused (which invokes the notion of ‘fetal personhood’), one could focus on the rights of the *future child* who, in any event would not be entitled to any compensation until he or she is born. Consider the following example:

*The future non‐lethal teratogen*: Company Z disposes of a toxic chemical that leaches out of its container and can affects fetuses five years later. The company is aware that this chemical is associated with a 25% chance of causing physical defects and brain damage to fetuses. Nevertheless, Z disposes of the chemical. Twenty‐five percent of local children (born after the chemical is released) are subsequently affected by physical defects and brain damage.^21^

Even if their action took place five years before the injury is suffered Company Z should be found guilty of a crime against the affected children. Although the children did not exist at the time the chemical was discarded, or were not even conceived, the company's actions affect these future people.

The concept of harm to the future child would provide a means for legal redress where a third party has caused non‐lethal in‐utero harm.^22^ But what difference should it make if the party causing harm is the pregnant woman?

## Gestational Harm and the Future Child

Where harm occurs after a child is born, the fact that it is the child's parent who has harmed them provides no reason for exemption from prosecution or compensation. (Indeed, from a moral perspective, we might think that a parent's special obligations to protect their child mean that it is worse for a parent to harm their child, than for a third party to cause the same harm).

*The non‐lethal gestational harm case*: A woman is aware that consuming a particular substance during pregnancy imposes a 25% risk of causing physical defects and brain damage to future children. Nevertheless, she elects to take the substance during pregnancy. Her child is subsequently affected by physical defects and brain damage, likely attributable to taking the substance.

If harm to the future child allows the possibility of legal action in third party cases, we might think that if the pregnant woman injures her fetus in‐utero, the fact that the fetus is not then a person should not bar the award of compensation for injuries to the child later. In the non‐lethal gestational harm case, where a pregnant woman ingests a toxin that she is aware will harm her future child, she should be held accountable for that wrong. For example, she might be charged with a criminal offence, or the child might be removed from her care. There could be compensation for a crime committed against the child before birth, if the public policy protecting a pregnant woman from liability for harming her fetus in‐utero was changed.

For compensation to be awarded for a pre‐birth injury caused by the mother, two alternatives could be considered – a civil claim against the mother and a claim against the state as a victim of a crime. Claims against the mother for compensation for a pre‐birth injury are not allowed by the law because they are potentially not in the child's best interests. If the mother is required to pay compensation to her child for a pre‐birth injury, that would reduce the funds available to the family and thus to the child, as well as causing tension between family members. There is a limited exception allowing the mother to be sued where the child's pre‐birth injury was caused by the mother's negligent driving of a car while she was pregnant. In that case, the compensation would be paid by an insurer and not come from family funds.^23^ However, as claims seeking compensation for being the victim of a crime are claims against the state, they are not open to the same public policy objections as a civil action against the mother (though there may be other objections to the state funding such loss).^24^ If compensation was awarded in such a case, that would seem to involve a finding that the pregnant woman had committed a crime and she could then be prosecuted. But like civil actions against the pregnant woman, such prosecutions might be considered contrary to public policy, pitting the pregnant woman's interests against those of the child and the family.

This situation was recently considered by the Court of Appeal for England and Wales.^25^ The question was whether a child (CP), who was diagnosed with serious harm from FASD, should be compensated for being the victim of a crime. The Criminal Injuries Compensation Authority provides financial support for people who have been innocent victims of violent crimes and have been harmed as a result. The local authority argued that the CP's mother was guilty of having ‘maliciously administered a poison’, (Offences Against the Persons Act 1861 (UK) s 23) resulting in grievous bodily harm to CP. In this case there was no dispute about whether or not CP's mother had drunk excessively prior to CP's birth, or whether CP had been seriously harmed as result. However, the Court dismissed the appeal, on the basis that the fetus was not a living person.

## Should the Law Recognize In‐Utero Harm?

Current law in countries including the UK and Australia may preclude either prosecution or compensation for pre‐birth injury after a child is born. However, it is worth distinguishing four separate arguments for avoiding legal action in such cases.

### Personhood: The fetus does not have the legal/ethical status of a person

As noted above, in the case of CP, the fetus was judged not to have been a ‘person’ at the time of the mother's actions, and therefore was judged ineligible for later compensation. A fetus, or a representative acting on behalf of the fetus, cannot commence legal proceedings regarding an injury in‐utero; the child must be born alive in order for compensation to be awarded for that injury.^26^ However, even if the fetus has no legal status at the time of an action, we have argued that harm to future children is morally significant. In those cases in which the fetus will become a child, there will be a child in the future for whose injury compensation and redress would be appropriate. In the future teratogen case, we would still wish to find Company Z guilty of a crime, even if no child affected had been conceived at the time of their action.

### Abortion: Granting the fetus legal status might impact upon abortion rights

If the fetus were granted personhood, a pregnant woman and medical professionals might be found guilty of causing the death of a person where a termination of pregnancy has taken place. Yet, the concept of future harm has no such implications. Harm to future children only occurs where there are or will be living persons with full moral status who exist in a worse state because of a prior event; i.e. children born alive who have incurred an injury in‐utero. It is therefore only applicable to non‐lethal in‐utero harm. Actions that lead to the death of a fetus (such as termination of pregnancy) would never lead to a person being harmed who unequivocally has full moral status. Indeed, recognition of future harm might be thought to represent a separate and important justification in support of access to termination of pregnancy. Some women may discover that they are pregnant in the first or second trimester, having already consumed significant amounts of alcohol, or other agents that risk harm to the future child. Women may choose to terminate their pregnancy in order to prevent the possibility that a child will be born who has been harmed by their actions.^27^

### Autonomy: Prevention of in‐utero harm might have unacceptable implications for the autonomy of pregnant women

In cases of gestational in‐utero harm, it is sometimes claimed that it is not in the ‘public interest’ to prosecute. One reason for this is the concern that preventing in‐utero harm might require major infringements of the liberty of pregnant women. In the US, pregnant women have been detained or had their behaviour restricted under a court order, to protect a fetus; and in the US and the UK, women have been compelled by court orders to undergo a caesarean section in the interests of the fetus.^28^ There have been no cases of forced caesareans reported in Australia, but, in a relatively recent case in the New South Wales Supreme Court on the refusal of medical treatment, the judge appeared to leave open the possibility that a court might override a woman's refusal of treatment to protect a fetus.^29^ ‘There may be a qualification [on the general need for consent]’ he said, ‘if the treatment is necessary to save the life of a viable unborn child’.^30^

However, there is a range of different possible actions that might be taken. For example, measures that might be considered to protect a fetus from later suffering from FASD are listed in the box below in order of increasing possible impact upon the pregnant woman's autonomy.

Box 2 **Possible pre‐natal interventions in response to gestational in‐utero harm from consumption of alcohol**

A1. Programmes to reduce drinking in the whole community
A2. Pre‐pregnancy education with a special focus on young women who drink heavily and warnings in pregnancy testing kits; (similar education campaigns have been successful for spina bifida and rubella)
A3. Increased access to termination of pregnancy for women who may have drunk heavily during pregnancy
A4. Voluntary counselling for pregnant women after pregnancy has been confirmed about safe levels of alcohol intake
A5. Encouragement of long‐term contraception for women known to be heavy drinkers (or even payment not to have a child)
A6. Mandatory reporting of suspected prenatal risks to a future child (similar to mandatory reporting of suspected child abuse)
A7. Mandatory counselling for pregnant women who have a history of risky drinking
A8. Measures to restrict alcohol sales or serving of alcohol to pregnant women (e.g. holding publicans responsible for serving a pregnant woman)
A9. Mandatory detention of the woman until the child is born
A10. Mandatory termination of pregnancy in cases of heavy drinking during pregnancy

In considering what types of intervention may be justifiable, there may be a distinction between negative and positive constraints on a pregnant woman's activities. The degree of ‘restraint’ that is justifiable will obviously vary according to the perceived risk and other relevant factors.

Some of the interventions listed in the table (A1‐A5) involve no limitations for the woman, and represent actions that are relatively uncontroversial and should be adopted. Others involve relatively small infringements of the woman's autonomy. Where it is possible to avert harm by taking actions that involve little or no self‐sacrifice, the ‘Duty of Easy Rescue’ implies that there is a strong ethical obligation to take that action.^31^

Consider the following analogy:

*Immunisation to prevent in‐utero harm*: Rubella, or German measles, is a benign disease, except if a woman contracts it when she is pregnant. It then causes congenital rubella in the fetus, potentially involving intellectual disability, blindness and deafness in the child after birth. Most people are vaccinated against rubella, not for their own benefit, but to protect future children. Imagine that rubella changes and the old vaccine is no longer effective. An epidemic emerges so that 1/100 babies are born with severe intellectual disability. But fortunately, a new safe vaccine is developed. Should it be mandatory for women of child‐bearing age to be vaccinated, so that millions of children could be spared severe intellectual disability?

The argument for mandatory vaccination in such a case is compelling. The injury is very serious; the risk that it will occur is high; many people will be affected; and the new vaccine would prevent it. However, despite a general policy in the community that vaccination should be voluntary perhaps the circumstances in this scenario are sufficiently different to justify mandatory vaccination? If there were other ‘easy rescue’ interventions that could prevent in‐utero harm, they should be considered. The harm to the pregnant woman imposed by interventions A6‐A8 may seem to be vastly outweighed by the potential harm to the future child that is prevented. We suggest that these interventions too should be adopted in order to prevent future harm.^32^

The other factor to take into account is the degree to which liberty‐restricting actions are justified to prevent the *possibility* of harm. In general, preventative detention (A9) (i.e. in order to prevent a crime from taking place) is usually thought to be justified only where there is a virtual certainty of harm occurring in the absence of detention. In a recent study, 52 women had a history of drinking at high risk levels, but only 13 children were diagnosed with FASD. A policy of mandatory detention would potentially affect a number of women whose children would not suffer major harm.^33^ In line with approaches to preventative detention elsewhere (for example, in the setting of mental illness),^34^ detention only represents an ethical alternative where all less‐restrictive options have been exhausted and there is a very high probability of significant harm to a future child.^35^

More intrusive interventions such as forced caesarean section or forced termination of pregnancy (A10) represent serious infringements of the rights of a woman. They might not be justified even in the setting of certain significant harm.

### Self‐defeat: Post‐harm legal options may be of no benefit to the child, or harmful to the child

If the mere possibility of harm is usually not sufficient to justify legal action, action may still be taken later if harm has occurred. In the case of CP, it was accepted that the child's mother had definitely consumed alcohol, and that CP had been harmed as a result. In other cases there may be difficult questions about whether harm resulted from maternal action or other environmental (or genetic) factors. There may be questions about intent.^36^ In such cases, it would seem difficult to prove the mental element required for criminal liability. As a commentator said in the news report about the CP case, ‘No mother deliberately holds a gun to their child's head’;^37^ and although reckless or gross negligence can be sufficient *mens rea*, even that level of culpability may be difficult to prove. In addition there may be other exculpatory factors that have led to alcohol or other addiction and warrant a lenient approach. However, there is a further practical concern that some actions taken after harm has occurred may appear self‐defeating (Box 3). For example, prosecution or incarceration of a mother for having consumed alcohol during pregnancy may risk separating a child from their parent, or preventing contact with their parent. Legal action to secure compensation might appear to be simply robbing Peter to pay Paul, where the mother is already financially supporting the child, or may be of limited practical value to a child because the mother has very limited financial resources.

Box 3 **Possible post‐natal legal actions after gestational in‐utero harm**

B1. Criminal prosecution of a mother for causing harm to her future child
B2. Criminal prosecution of a third party for contributing to, or failing to prevent in‐utero harm
B3. Compensation from a mother for having caused harm
B4. Compensation from a third party for having caused or contributed to harm

Yet neither of these factors will always rule out post‐harm legal action. Children who have suffered gestational in‐utero harm may no longer be in the care of their mother and may have limited existing contact. In such situations, imprisonment of the mother would not necessarily lead to additional harm to the child. Equally, the self‐defeating objection would not rule out compensation from other sources. For example, in the case of CP, the compensation sought was from a nationally funded programme for the victims of violent crime, and would appear to offer genuine benefit to the child. Alternatively, if a child sought compensation from another source (for example, from companies who sold or marketed alcohol to his pregnant mother), the self‐defeat objection would not apply.

## Conclusion

FASD and other fetal conditions that may be caused by a pregnant woman's risk‐ taking behaviour may result in serious and preventable harm to the child. Under the current law in countries including the UK and Australia, a fetus is not legally a person and no one can bring legal proceedings on the fetus’ behalf to protect him or her from harm in‐utero, or to compensate the child for in‐utero injury until after the child is born. In most societies, a pregnant woman's right to autonomy and freedom of action will prevail over rights that might be argued on behalf of the fetus.

Legal intervention to restrict a pregnant woman's activities in order to protect the fetus could be seen as conferring the status of ‘personhood’ on the fetus and have serious implications for the law of abortion. However, we have argued that regarding a fetus’ rights from the perspective of the future child would enable compensation to be awarded after birth for pre‐birth injuries without denoting fetal personhood.

In cases of gestational harm, not all legal protective measures that might be possible are justifiable or practicable. The measures that are most likely to be successful and widely supported are education, support and counselling. These could perhaps be mandatory as the interests of the unborn or future child that have been discussed in this article would arguably support that level of ‘duress’ of the pregnant woman.

While we have focussed on in‐utero harm, our arguments apply to actions taken around conception, or even before conception, which manifest themselves as making a child in the future worse off than she or he would have been if those actions had not been performed. Moreover, if one rejects the moral distinction between acts and omissions, the same arguments apply to omissions before or after conception that foreseeably and avoidably leave a future child worse off. For this reason, it is ethical to provide compulsory fortification of cereals with folate to prevent spina bifida.

Although he or she does not currently exist, the future child should be of concern to us now, and, where necessary, should be recognized by the law. Although this question often seems overshadowed by disagreements over abortion, it need not be. Pro‐choice and pro‐life can agree on this: our ethical obligations to future children are significant. Just as for our existing children, the obligation to prevent harm to future children may require significant sacrifices on the part of parents, prospective parents, and wider society.

## Footnotes

1

J. Savulescu . Future People, Involuntary Medical Treatment in Pregnancy and the Duty of Easy Rescue. Utilitas 2007; 19 (1): 1–20.

2

National Centre on Birth Defects and Developmental Disabilities . 2014 Birth defects: Data and statistics. Atlanta. Available at: http://www.cdc.gov/ncbddd/birthdefects/data.html [accessed 8 Apr 2015].

3

H. Dolk . Epidemiologic approaches to identifying environmental causes of birth defects. Am J Med Genet C Semin Med Genet 2004; 125C: 4–11. 1475542810.1002/ajmg.c.30000

4

ACOG Committee Opinion No. 524: Opioid abuse, dependence, and addiction in pregnancy. Obstet Gynecol 2012; 119: 1070–1076. 2252593110.1097/AOG.0b013e318256496e

5

National Centre on Birth Defects and Developmental Disabilities . 2014 Facts about FASDs. Georgia. Available at: http://www.cdc.gov/NCBDDD/fasd/facts.html [accessed 8 Apri 2015].

6

P.W. Kodituwakku . Defining the behavioral phenotype in children with fetal alcohol spectrum disorders: a review. Neurosci Biobehav Rev 2007; 31: 192–201. 1693070410.1016/j.neubiorev.2006.06.020

7
FASD is the leading cause of avoidable intellectual disabilities in the US ( S.E. Maier & J.R. West . Drinking patterns and alcohol‐related birth defects. Alcohol Res Health 2001; 25: 168–174. 11810954PMC6707176 A 1995 study estimated a FASD global incidence of 0.97 per 1000 live births. E.L. Abel . An update on incidence of FAS: FAS is not an equal opportunity birth defect. Neurotoxicol Teratol 1995; 17: 437–443. 756549010.1016/0892-0362(95)00005-c

8

P.A. May , et al. Prevalence and epidemiologic characteristics of FASD from various research methods with an emphasis on recent in‐school studies. Dev Disabil Res Rev 2009; 15: 176–192. 1973138410.1002/ddrr.68

9

J.P. Fitzpatrick , et al. Prevalence of fetal alcohol syndrome in a population‐based sample of children living in remote Australia: The Lililwan Project. J Paediatr Child Health 2015; 51: 450–457. 2559424710.1111/jpc.12814

10
Maier *op. cit*. note 7.

11

L.M. O'Keeffe , R.A. Greene & P.M. Kearney . The effect of moderate gestational alcohol consumption during pregnancy on speech and language outcomes in children: a systematic review. Syst Rev 2014; 3: 1. 2438342210.1186/2046-4053-3-1PMC3892059

12
A child can claim compensation for a pre‐birth injury under the Congenital Disabilities Act 1976 only if born alive.

13
Ibid. This occurred in the thalidomide cases, for example, where the children were eventually compensated for the injury the drug caused to them. For a recent article on thalidomide, see H. Evans. 2014 Thalidomide: how men who blighted the lives of thousands evaded justice. *Guardian* 14 November 2014. Available at: http://www.theguardian.com/society/2014/nov/14/-sp-thalidomide-pill-how-evaded-justice [accessed 8 Apr 2015].

14
2013. Crimes amendment (Zoe's law) bill (no. 2). Available at: http://www.austlii.edu.au/au/legis/nsw/bill/calb22013293/ [accessed 8 Apr 2015].

15

2011 Woman jailed for crash that killed unborn baby. ABC News. Available at: http://www.abc.net.au/news/2011-03-31/woman-jailed-for-crash-that-killed-unborn-baby/2633524 [accessed 8 Apr 2015].

16

L. Blayden . 2013 Crimes Amendment (Zoe's Law) Bill 2013 (No 2), NSW Parliamentary Library Briefing Paper, e‐brief 8/2013. Available at: http://www.parliament.nsw.gov.au/prod/parlment/publications.nsf/key/CrimesAmendment%28Zoe%27sLaw%29Bill2013/$File/Crimes+Amendment+%28Zoe%27s+Law%29+Bill+2013.pdf [accessed 8 Apr 2015]. A criminal charge is, of course, different from a claim for compensation for injuries arising from alleged negligence but both raise the issue of the fetus being regarded as a separate person from the mother – as the victim of a criminal offence or the claimant in a civil action.

17
There was an exception in the Bill for acts undertaken during a medical procedure (covering abortion) or with the mother's consent. However, this did not prevent concerns about its implications for the law of abortion.

18
The Bill for Zoe's law was supported by a number of prominent Roman Catholic bishops for its view that unborn children are persons and opposed by women's groups concerned about its implications for women's reproductive choices. B. Jabour. 2014. Catholic bishops urge NSW politicians to vote in support of Zoe's law. *Guardian* 16 March 2014. Available at: http://www.theguardian.com/world/2014/mar/16/catholic-bishops-urge-nsw-politicians-to-vote-in-support-of-zoes-law [accessed 8 Apr 2015].

19

I. Karpin . Legislating the female body: reproductive technology and the constructed woman. Columb J Gend Law 1992; 3: 325–349.

20
Children's actions for compensation for their own injuries (so‐called ‘wrongful life’ cases) have been rejected in a number of Australian cases: eg Alexia Harriton, who was born blind, deaf, spastic, intellectually disabled after her mother's rubella was not diagnosed during pregnancy: *Harriton v Stephens* [2006] HCA 15; Chelsea Edwards, who was born with cri du chat syndrome after a failed vasectomy: Edwards v Blomeley [2002] NSWSC 460 (12 June 2002); and IVF baby Keeden Waller, who was born with a clotting disorder AT3 inherited from his father which could have been detected by fetal test: Waller v James;Waller v Hoolahan [2006] HCA 15. There is an extensive literature on such cases, with some commentators arguing that compensation should be payable directly to the child rather than indirectly to the parent. For examples of commentary, see McGivern, Brenda; Ellis, Evelyn, ‘The wrongfulness or rightfulness of actions for wrongful life’ (2007) 15 Tort Law Review 135‐161; Stretton, Dean, ‘Harriton v Stephens; Waller v James: Wrongful Life and the Logic of Non‐Existence’ (2006) 30(3) Melbourne University Law Review 972. In the UK, the Congenital Disabilities (Civil Liability) Act 1976 deprives children born after 22 July 1976 of any possible action for ‘wrongful life’: McKay v Essex AHA [1982] QB 1166 (per Ackner LJ, at 1186‐7). Note that the idea that this statutory law deprives children of an action for wrongful life has been challenged in the academic literature (see J Fortin, ‘Is the “Wrongful Life” Action Really Dead?’ (1987) *Journal of Social Welfare and Family Law* 306–313).

21
We should note that some ‘future harm’ cases are identity affecting: D. Parfit . 1984 Reasons and persons. Oxford: Oxford University Press pp. 355–6. As a consequence of the action taken, the timing of conception may change, and different children may result. In such a situation, it is less plausible to claim that a future person is harmed. C. Foster , T. Hope & J. McMillan . Submissions from non‐existent claimants: the non‐identity problem and the law. Med Law 2006; 25: 159–173. Such cases are beyond the scope of this article. 16681120

22
It would not address the specific situation in Zoe's law, where the action of a third party leads to a fetal death.

23
Watt v. Rama [1972] VR 353.

24
In the UK, there have been recent moves to reduce the amount of compensation paid by the state (see, e.g., D. Miers , ‘Compensating Deserving Victims of Violent Crime: the Criminal Injuries Compensation Scheme 2012’ (2014) 34(2) Legal Studies 242, at 250).

25
Re CP (A Child) [2014] EWCA Civ 1554.

26

P. Stewart & A. Stuhmcke . Legal pragmatism and the pre‐birth continuum: an absence of unifying principle. J Law Med 2007; 15: 272–295. see also Watt v Rama *op. cit*. note 23 at 360 (per Winneke CJ and Pape J); Lynch v Lynch (1991) 25 NSWLR 411. 18035844

27
Policies to increase access to termination of pregnancy, or decisions to terminate pregnancies might be motivated by a desire to avoid future harm, or by a desire to avoid future liability. The latter might be of particular concern to those who are opposed to abortion.

28
There have been a number of such cases in the US and the UK (but not in Australia): In Re S (Adult: Refusal of Treatment) [1992] 3 WLR 806; in re Madyun, Reprinted in (1990) 573 A 2d 1259 as an Appendix to In Re AC 573 A 2d 1235; Jefferson v Griffin Spalding County Hospital Authority (1981) 274 SE 2d 457; see alsoVEB Kolder; J Gallagher; MT Parsons, ‘Court‐ordered obstetrical interventions’ (1987) 316 New England Journal of Medicine 1192; Les Haberfield, ‘Pregnant women: Judicial intervention and the right of pregnant women to refuse medical treatment’ (1995) JCULR 1. However, this has not been the line taken in the later cases in England and Wales such as St George's Health Care NHS Trust v S [1998] 3 WLR 936. More recently, courts have increasingly recognized a pregnant woman's right to refuse medical treatment; eg. In re AC 573 A2d 1235 (1990 DC App).

29
Hunter and New England Area Health Service v A [2009] NSWSC 761 (6 August 2009).

30
Ibid, para 40(1) (per McDougall J). One of us (Julian Savulescu) has argued that the issue of saving a fetus' life is different from the issue of preventing avoidable, foreseeable future harm and that regardless of the moral status of the fetus, prevention of fetal harm is a moral imperative. J. Savulescu , Future people, involuntary medical treatment in pregnancy and the duty of easy rescue, Utilitas 2007; 19 (1):1–20.

31
Savulescu, *op. cit*. note 30.

32
There are practical and ethical issues to address with each of these potential policies. For example, restrictions on serving alcohol may be ineffectual early in pregnancy (when pregnancy is not apparent), and may risk causing cause offence to women who are suspected of being pregnant when they're not. Such issues are beyond the scope of this article.

33
Fitzpatrick *op. cit* note 9. It is of course possible that children born in the setting of high risk alcohol intake may be impaired, even if they do not meet the criteria for a diagnosis of FASD. Savulescu *op. cit* note 1.

34

G. Szmukler & P. Appelbaum . Treatment pressures, leverage, coercion, and compulsion in mental health care. J Mental Health 2008; 17: 233–244.

35
Two further potential objections to detention during pregnancy might also have parallels with detention for mental health reasons. Some may have concerns that such a policy would disproportionately affect women from poorer socio‐economic groups, or that it would discourage women from seeking antenatal care. E. N. Linder . Punishing prenatal alcohol abuse: the problems inherent in utilizing civil commitment to address addiction. Ill Law Rev 2005; 3: 875–6. While coercive treatment of the mentally ill might lead to healthcare avoidance, this is typically balanced against the benefits of treatment, and is thought to nevertheless permit coercion or detention in some cases. The principle of non‐discrimination means that women should not be assumed to be consuming alcohol because of their socio‐economic background. However, if excessive alcohol consumption disproportionately affects women of poor socio‐economic background, this may provide extra reason to intervene (since future children will have compounded disadvantage). Higher rates of serious mental illness amongst those from deprived backgrounds is not typically thought to invalidate the case for compulsory treatment in the most severe cases.

36
For example, a woman should not be held accountable for acts or omissions that harm her fetus in‐utero if she does not know that she is pregnant. If legal action were allowed against a pregnant woman for wrongfully causing injury to her fetus in‐utero, it may be challenging to prove that she knew she was a pregnant if the woman does not utilize any healthcare service.

37

D. Howell . 2014 Pregnancy drinking examined as possible crime in landmark case. BBC News 5 March 2014. Available at: http://www.bbc.co.uk/news/uk-26031422 [accessed 8 Apr 2015].

